# Highlights from the 7th Oncological Pathology Conference ‘Pathological Anatomy in the context of the National Cancer Law: An overview of the Latin American experience’, 15, 22 and 23 July 2022, Trujillo, Peru

**DOI:** 10.3332/ecancer.2022.1462

**Published:** 2022-11-02

**Authors:** Caddie Laberiano-Fernández, Joan Moreno Luján, Bruno de Carvalho Dornelas, Magali Franco Benites, Patricia Gutiérrez Quispe, Valeria Aguilar Vásquez, Andric Guerrero Espinoza, Elsa Guerra Guerra, Gabriela Gil-Arroyo Álvarez, Juan Astigueta-Pérez, Maria Teresa Garcia de Dávila, Sandro Casavilca Zambrano, Tatiana Vidaurre Rojas, Alejandro Mariños, Emmanuel S González, Rossana Lazcano, Ricardo R Lastra, Isabel Alvarado-Cabrero, Henry Guerra Miller, Ricardo H Bardales, Milagros Abad-Licham

**Affiliations:** 1 International Academy of Cytology, 79098 Freiburg, Germany; 2 Peruvian Society of Medical Oncology, Lima 15037, Peru; 3 Clinical Hospital of the Federal University of Uberlandia, Uberlândia, MG, 38405-320,; 4 Ramiro Prialé Prialé National Hospital, Huancayo 12006, Peru; 5 Carlos Alberto Seguín Escobedo National Hospital, EsSalud, Arequipa 04001, Peru; 6 Northern Regional Institute of Neoplastic Diseases, Trujillo 13008, Peru; 7 Alberto Sabogal Sologuren National Hospital, Callao 07011, Peru; 8 Regional Hospital of Lambayeque, Chiclayo 14012, Peru; 9 Antenor Orrego Private University School of Medicine, Trujillo 13008, Peru; 10 Garrahan and British Paediatric Hospital of Buenos Aires, Buenos Aires C1245 CABA, Argentina; 11 National Institute of Neoplastic Diseases, Lima, Surquillo 15038, Peru; 12 MD Anderson Cancer Center, Houston, TX 77030, United States; 13 Dr. Enrique Baltodano Briceño Hospital (HEBB), CCSS, Liberia 50101, Costa Rica; 14 The University of Chicago Medical Center, Chicago, IL 60637, United States; 15 Star Medica Central Hospital, Mexico; 16 Mexican Oncology Hospital, Mexico City 14080, Mexico; 17 Outpatient Pathology Associates/Precision Pathology, Sacramento, CA 95826, United States; 18 Centre of Excellence in Pathological Oncology, Trujillo 13011, Perú; a https://orcid.org/0000-0003-4513-6123; b https://orcid.org/0000-0003-2621-7198; c https://orcid.org/0000-0003-1404-8876; d https://orcid.org/0000-0002-4872-1646; e https://orcid.org/0000-0002-1491-1556; f https://orcid.org/0000-0001-6889-0175; g https://orcid.org/0000-0002-2619-1920; h https://orcid.org/0000-0002-6320-1278; i https://orcid.org/0000-0002-6396-999X; j https://orcid.org/0000-0001-5984-3270; k https://orcid.org/0000-0002-3561-5035; l https://orcid.org/0000-0001-8406-739X; m https://orcid.org/0000-0003-1995-4560; n https://orcid.org/0000-0001-8179-5789; o https://orcid.org/0000-0001-6204-3231; p https://orcid.org/0000-0001-9890-2325; q https://orcid.org/0000-0003-0691-5685; r https://orcid.org/0000-0003-4000-9280; s https://orcid.org/0000-0002-4894-5631; t https://orcid.org/0000-0003-1238-8535; u https://orcid.org/0000-0002-3530-6937

**Keywords:** cancer, pathology, oncology, Peru, Latin America

## Abstract

The seventh session of the Oncological Pathology Conference (JoPaO) entitled ‘Pathological Anatomy in the context of the National Cancer Law: An overview of the Latin American experience’, was held virtually on July 15, 22 and 23. Peru was the headquarters for this event, where 17 national and international professors of high academic standing participated. They interacted in a multidisciplinary context through talks with national panellists and the general public. The recent promulgation of the ‘National Cancer Law’ fosters the development of discussion forums to analyse the national realities and uphold continuous learning about experiences in other Latin American countries with successful cancer programmes, in which pathology holds a principal role. The topics addressed during this JoPaO included the exchange of Latin American cancer management experiences, an emphasis on investments in and the development of strategic plans to improve care, the use of new technologies, laboratory quality control, and the need to advance scientific research.

## Introduction

In 2010, the first Oncological Pathology Conference (JoPaO) was held in Trujillo, Peru, under the slogan: ‘A Pathology-Centred Multidisciplinary Approach to Oncology’. Over the years, this has subsequently become a Latin American model for multidisciplinary conferences.

On July 15, 22 and 23, 2022, the seventh session of this conference was held virtually owing to the COVID-19 pandemic-related restrictions. However, its essence was maintained by taking as its basis the dynamic interaction between pathology and other medical specialities, which strive for appropriate management of the cancer patient.

This virtual platform facilitated the participation of several renowned national and international speakers. It also enabled their interaction with more than 200 registered participants. In total, ten international and seven national professors took part. Seventeen talks were thereby held, in which they shared their knowledge and experience. This event was sponsored by the country’s main professional associations as well as renowned national and Latin American scientific societies.

The central topic of this conference was the role of pathological anatomy under the National Cancer Law in Peru, for which the legal scope was set out in recently passed Law N°31336. This law seeks to ensure universal, free, and priority health coverage for all cancer patients, thus safeguarding health as a fundamental right on equal terms without discrimination [1].

Also, a discussion was held on the similarities and differences in cancer-related care between the various countries of this region. It was concluded that analysis was required not only from a medical perspective but also from a social and economic perspective. In this respect, experts shared their experiences from three perspectives: the role of the state, the laboratory, and the multidisciplinary team. Finally, the functionality of these domains was compared with those of other countries to reach a consensus with the objective of improving cancer care.

## Scientific talks

### • Cancer in Peru: an insight into where we are at present

Dr. Gustavo Sarria, who works in the National Institute of Neoplastic Diseases (INEN), Peru, began the keynote presentations by providing a comprehensive insight into the cancer situation in this country. Life expectancy in Peru and Latin America at birth has increased by 20 years over the last five decades. This increase in longevity will significantly impact the healthcare systems. Consider the case of Peru, where the cancer incidence is expected to rise from 70,000 new cases a year to 4,351,140 by 2060 if there is no intervention. This significant increase makes cancer a serious public health problem not only because of the immense number of patients affected, but also because it has been the leading cause of premature death since 2018.

Two critical problems in the Peruvian health system are the fragmentation at the national level and the centralisation of oncology specialists in the capital, limiting the capacity for early diagnosis. Therefore the need to create a more articulated health system. He noted that by not improving the decentralisation of cancer management, the health system would collapse, thus worsening the already outstanding social debt. In his view, the recent National Cancer Law has some original vital points, including better organisation of the health system, infrastructure investment advocacy, and the creation of epidemiological records.

Finally, he also explained that a financial effort on the part of the public sector was required and proposed three ways to make progress towards this universal health coverage: increase the insured population, reduce the co-payment of diagnosis and/or treatment-related costs and include other services.

### • Pathology laboratories and the National Cancer Law

Dr. Sandro Casavilca, who works in INEN, Peru, presented a review of the National Cancer Law, which highlighted the recent inclusion of advocacy, control, prevention, and cancer care services. He also noted the benefits that come with the creation of the National Network of Oncology (RON in Spanish). This effort would be led by the National Institute of Neoplastic Diseases (INEN) in its capacity as a public health institution, which is comprised of various regional cancer institutes and centres, with the objectives of providing adequate diagnoses, timely care, and follow-up for cancer patients on a national scale [1].

He emphasised that the RON had the following mission processes: a) Management of Health Benefits, b) Management of Cancer Epidemiological Monitoring, c) Management of Cancer Research, d) Management of Technology Transfer and Standardisation and e) Monitoring and Evaluation [1].

In this regard, Dr. Casavilca underlined the role that pathological anatomy fulfils in: the diagnosis, prognosis, and staging; prediction of the response and the assessment of treatment efficacy; the epidemiological record and research; and the creation of tissue repository centres. He, therefore, believes that pathologists and clinical oncologists should collaboratively search for more effective treatment options by adopting precision medicine and companion test approaches [2–5], molecular methodologies, and networked digitised imaging systems that use `big data' methods to foster computational pathology development [6].

Cancer heterogeneity verification, biomarker diversity, and differences in treatment responses have led to extensive efforts to better define the characteristics of each cancer at the histological and molecular levels. As such, today’s pathologists must identify molecular alterations and interpret them in the appropriate morphological context, consider the pre-analytical variables, calculate the proportion of tumour cells in the sample submitted for analysis, evaluate the presence of inflammatory infiltrates and necrosis, select the most appropriate further research methodologies and control the comprehensive diagnostic molecular results turnaround time [3, 7].

He ended this session by discussing the bid to develop a diagnosis support system, enabling us to improve the health programmes combatting cancer. This will require a strategy of harmonisation and governance led by INEN, geared towards optimising resources as per the level of care complexity and the underlying referral system. Likewise, he underlined the implicit prioritisation of technical support and technology transfer in developing local needs-based regional plans [1, 8].

### • Proposals for better cancer diagnostic and therapeutic performance in Latin America

Dr. Isabel Alvarado, who works in Hospital Star Médica Centro, México and Hospital de Oncología, México, spoke about the cancer situation in this region, emphasising how it was a costly disease that will have a dramatic increase in incidence. Morbidity and mortality are the immense resulting costs of inaction, which must be considered when proactively facing the cost of cancer control programme implementation. She highlighted that high-income countries conduct more cancer research than low- and middle-income countries (LMIC) [9].

From an overall perspective, there are significant challenges concerning cancer diagnosis, the increased access needs of patients, the requirement for highly trained staff, the optimisation of ongoing equipment performance and maintenance, as well as the steady distribution of supplies and reagents [10]. Moreover, she noted the importance of early detection, which improves the survival outcome for patients by increasing treatment options, prolonging survival, and improving quality of life. However, financial restrictions and administrative, and political decisions have an impact on cancer control, efficacy, and the inclusion of overall cancer care [11].

National cancer data is a prerequisite for assessing the magnitude of this disease burden and an essential criterion for evaluating the efficacy or inefficacy of any primary, secondary, or tertiary intervention. However, the poorly defined incidence, mortality, and cancer staging in many LMICs are among the issues we must address in our region. Another challenge is the delayed delivery of cancer diagnostic reports, which are dependent upon several interconnected services. The delivery of results should therefore be prioritised effectively as an integral part of comprehensive cancer control.

Dr. Alvarado reaffirmed the need to bolster cancer research. She mentioned that the Global Forum for Health Research defines the ‘10/90 gap’ as the fact that less than 10% of the global health research expenditure is dedicated to diseases and illnesses, which account for 90% of the worldwide morbidity burden. The impact of this gap is greatest in LMIC. She emphasised that research is essential in identifying effective intervention opportunities in areas in need. It also makes it possible to focus on health policies, gain investments and provide skill-building opportunities, which are critical components in sustainable service development [12].

Public policy is very influential in health care, including how pathology departments can be managed. The lack of leadership in pathology societies have characterised the debate on national and global health policies.

She ended her presentation by indicating the research priorities for LMICs over the next 10 years [13]:

- Reduce the load of patients who have advanced-stage disease;
- Improve access, affordability, and outcomes in cancer care through solution-oriented research;
- Emphasise the national health economic assessment, focusing on interventions and technologies related to cancer management, value-based mechanisms for financing health and care;
- Expand improvement in quality and the implementation of research in controlling cancer;
- Avail of the technology to improve cancer control with the support of solid scientific evidence.

### • Role of scientific societies within the framework of the National Cancer Law

The talk was given by Dr. Vidaurre, who works in INEN, Peru, who started by indicating that in Peru, there are around 50 medical societies endorsed by the Medical College of Peru [14], of which 16% are dedicated to oncological diseases, and the oldest is the Peruvian Society of Cancerology [15].

Oncological academic societies group together doctors from different oncological specialities and specialities related to cancerology, which seek to encourage professional development, the transfer of knowledge, research of scientific and social value, innovation, and technological development, in addition to patient protection through good oncological practice.

She also highlighted that the primary technical responsibilities of the societies are scientific exchange, continuous medical education, technical assistance to health bodies in the country, and the formulation, implementation, and dissemination of public policies on cancer prevention and control. Likewise, within the current role of academic societies, she highlighted: Health education and promotion, cancer prevention and patient-focused comprehensive care and community health, significant social participation, and the development of health management tools. These responsibilities seek to improve quality standards in providing oncological services and collaboration in exchanging scientific knowledge nationally and globally towards personalised and precision oncology.

Dr. Vidaurre concluded that under the National Cancer Law, academic societies would play a key role in activities such as prevention actions and early detection of cancer nationally and so contribute to incentivising and promoting health based on scientific evidence. Conversely, she stressed the importance of permanent and continuous training of medical specialists in oncology; therefore, participation at conferences, scientific seminaries, and educational activities must be promoted.

### • Paediatric tumour bank: the Argentinian experience

Dr. María Teresa García de Dávila, who works in Hospital de Pediatría Garrahan y Británico de Buenos Aires, Argentina, shared her experience in creating the paediatric tumour bank in her country, highlighting the scientific and social importance of biobanks, which have grown exponentially due to advances in biotechnology and translational medicine [16, 17].

The interdisciplinary character of biobanks creates opportunities for biomedical researchers, doctors, and industry study to collaborate and discover new prognostic factors, such as genetic mutations, moving forward in the field of personalised medicine [18, 19].

Dr. García de Dávila stated that the tumour banks in her country were started on 14 February 2005 in the pathology department of the Hospital Nacional de Pediatría Prof. Dr. Juan P. Garrahan. The official inauguration would be later on 11 April 2007 [20, 21].

In 2009, the group led by Garrahan was invited to participate in the meetings of the stem cell committee, and in 2011 to the meetings of the ad-hoc Biobank Committee of the Ministry of Science, Research and Technological Innovation (MINCYT). They worked on the drafting of recommendations for biobanks/biological resource centres. As members of the MINCYT Committee, they participated in different meetings throughout Argentina and Latin America, presenting the results of the committee in areas such as informed consent, use of samples, infrastructure, minimum requirements, patient rights, legislation and ethics in biobanks, to name a few [22–25].

In 2018, an inter-ministerial committee was established with the participation of the MINCYT and the Ministry of Health. This committee reviewed and extended different aspects and critical areas of the recommendations for biobanks in national research, serving as a guide in the absence of national regulations. On 29 December 2020, the biobank guidelines for biological samples of human origin for research were approved by the Ministry of Health by means of Resolution 2020-2940-APN-MS [20–24, 26–29].

The main success of this tumour bank was the quality of the samples that were used in research, with the outcomes on retinoblastoma and neuroblastic tumours published in international journals that had a strong scientific and academic impact. As a model for quality, Dr. de Dávila said that this biobank could be linked to national or international biobank networks to share experiences and benefit children with cancer by improving knowledge and therapeutic strategies in paediatric oncology.

### • Understanding quality in the oncological pathology laboratory

Dr. Carlos Barrionuevo, who works in INEN, Peru, started his talk by defining quality as the degree to which a set of characteristics meet pre-established requirements. In oncological pathology, this is measured according to the pathology report, which must be timely, accurate, and complete; therefore, the clinical-surgical team must be familiar with the written information, which is crucial when making therapeutic decisions.

He also pointed out that the institutional adoption of a quality management system makes it possible to set out its policy and objectives, and it is a determining factor in improving competence and development.

He also mentioned that certification is the procedure whereby a body ensures that a product, process, management system, or service meets the specified requirements. He commented that the main benefits of becoming certified are: Increase in competitivity, improvement of processes, access to public tenders, and compliance with legal requirements.

He added that accreditation is the process by which a technical authority recognises that an organisation is competent to execute specific activities, such as essays, calibrations, and medical analysis. Peru has the National Institute for Quality as its accrediting body, which promotes and ensures compliance with the National Policy on Quality [30].

The quality in pathology monitors five stages: pre-analytic, analytic, post-analytic, waiting period, and clinical satisfaction, the first three being particularly relevant. He highlighted that in the pre-analytic phase, the errors occur mainly in the fixation, the identification of the specimen, and the incomplete clinical history, having to adopt some strategies to minimise them, such as identification and labelling of the sample.

Additionally, control methods should be used in the analytic phase to improve diagnostic effectiveness, such as peer review, the correlation between frozen section and paraffin section, the correlation between cytology and surgical pathology, revision of previous diagnoses, and revision of cases in other hospitals. Finally, in the post-analytic phase, there might be errors in transcription, correction, verification, or validation of the report; therefore, it is recommended to obtain IT systems with platforms for its elaboration.

### • Better pathology practices for the analysis of genomic profiling

Dr. Felipe D'Almeida, who works in Hospital A. C. Camargo, Brazil, pointed out that molecular testing is key to accessing innovative therapies in the age of precision oncology. For this reason, the role of the pathologist in a molecular pathology laboratory lies in rating samples for testing, confirming that the tissue received matches the clinical scenario, verifying that the requested test is appropriate for each situation, ensuring quality control, and assessing the final report.

He pointed out that the appropriate samples can be obtained through resection, small biopsies, fine needle aspirations (formalin-fixed paraffin-embedded (FFPE) cellblock), liquids and organic effusion [31]. He suggested that for molecular testing, the paraffin block and a haematoxylin and eosin (H&E) stain sheet must be sent whenever possible. If sheets are sent, ten sheets must be sent without stains, sliced to 4 -5 microns of thickness to obtain a tissue volume of 1 mm^3^. The minimum cellularity of the required sample is recommended to be 20% of tumour cells. Paraffin blocks are preferred compared to non-stain sheets [31].

He also recommended that the tissue should be fixated with formalin and added to paraffin, using standard methods of fixation to preserve the integrity of the nucleic acids (fixation with neutral formalin at 10% for 6 -72 hours) and not decalcifying the sample [32, 33].

The most common problems of the core biopsy in the quantity and quality of the tissue occur due to the presence of clots, plenty of inflammatory tissue with scarce tumour, significant necrosis, and small specimens [34].

When core biopsies are made through interventional radiology, he recommended doing three or more biopsies using needles of 18G to 20G [35]. He also highlighted the importance of limiting the use of Immunohistochemistry (IHC) and preserving more sample for the molecular tests for the diagnosis (TTF-1 and p40, in lung, for example), therefore he recommended slicing 15 sheets: 1 for H&E, 1 for PD-L1, 4 for IHC, and 10 for molecular testing [36].

Finally, as regards the processing of the tissue, he suggested avoiding FFPE tissue exhaustion in the first sectioning as well as avoiding contamination, since the *floaters*, for example, are a potential source of diagnostic error.

### • Cervical cancer (CC) and the quality of the cytology laboratory

Dr. Carla Molina, who works in Facultad de Medicina, Universidad de Chile, gave a presentation about the Programme of Cervical cancer in Chile and its quality control. She mentioned that since 1987, government guidelines were established to execute cervical cytology in women 25 -64 years old, reaching 80% of the target population. Afterward, in 1993, External Quality control was started in cytology laboratories, which allowed the mortality rate for CC to decrease, even though the coverage has lowered to 40% in the last few years.

She pointed out some significant milestones in her country; for example, in 2014, the vaccine against human papillomavirus. (HPV) was added to the National Programme of Immunizations for girls between 9 and 10 years old, and 5 years later (2019), boys of the same age group were included, and the molecular detection of HPV as a screening method was initiated in the Detection Programme of Cervical Cancer. Annually, in the Chilean public sector, approximately a million annual cytologies are assessed in 22 public laboratories; however, in the last years, there has been a decrease of 45.4% compared to the year 2019, probably due to the COVID-19 pandemic. The presenter also commented on the change of the primary screening in Chile, where molecular techniques have been implemented to detect HPV, which will allow to detection of an additional 10%–15% of positive cases.

For the quality control of the Programme, an annual internal and external assessment is carried out. The ongoing improvement of the Programme includes internal quality control (re-screening, hierarchical screening, monitoring of cytological diagnoses, cytology-histology correlation, revision of false negatives), process control (sample taking and staining), and ongoing education about external quality control.

Finally, she pointed out that the Programme of Cervical Cancer Detection aims to assess the individual diagnostic abilities of the professionals participating in gynaecologic cytology and promote ongoing education. The strengths of this programme include having well-trained staff; the terminology uses unified comparable governmental codes to the Bethesda System, and the programme uses the CITOWeb software.

### • Molecular biology in the context of CC

Dr. Emmanuel Gonzales, who works in Hospital HEBB, CCSS, Liberia, Costa Rica, mentioned that out of ten women who die of CC in the world, nine are LMIC, which reflects the clear inequality in health systems [37].

Pathophysiologically, HPV initially creates a proliferative, infectious self-limiting disease that conditions morphological changes of lowgrade lesions. Afterwards, it can develop into a transforming illness caused by a control breakdown between the viral genome expression and the epithelial differentiation, favoring an overexpression of viral oncogenes and, consequently, a clonal proliferation of undifferentiated cells. Furthermore, high-risk HPV (HR-HPV) can cause variations in E6 and E7 oncoproteins that bring about oxidative stress and favour the integration of viral DNA in the host cell DNA, thereby transforming the DNA and precancerous lesions.

He also pointed out that cervicovaginal cytology has some limitations, such as scant sensitivity for high-grade squamous intraepithelial lesions (HSIL), variable results between different laboratories, and deficient detection of adenocarcinoma [38, 39]. Likewise, according to data from the ESTAMPA study [40], an observational study that included around 50,000 women at 11 sites in Latin America, Pap smear test (PAP) sensitivity was situated between 48.51% and 56.27%; however, specificity is around 96%; therefore, Dr. Gonzales thinks that the PAP should be considered as a secondary triage test for HPV-positive patients.

Currently, the primary screening options are the HPV test (partial genotyping), the HPV test with cytology (co-testing), and PAP. However, according to the College of American Pathologists and American Society of Colposcopy and Cervical Pathology for primary CC screening, molecular methods must be used to detect HPV infection [41]

Among the available HPV tests, there are some methods such as hybrid capture, an old test for diagnosing viral persistence that does not recognise types of HR-HPV, it is used as a mirror for atypical squamous cells of undetermined significance or screening along with cytology, and continuous colposcopy studies are required to achieve a positive result. Moreover, two PCR platforms are approved by the U.S. Food and Drug Administration.

He ended by saying that the most significant HPV biomarker is p16, whose use is recommended when H&E produces uncertainty between HSIL versus simulator and in the instance of disagreement between pathologists [42]. Other biomarkers in cytology are dual cytology. (detecting p16 and Ki-67 in the same cell [43] and the mRNA E6, E7 test.

### • Let’s talk azbout quality, let’s talk about IHC

Dr. Lazcano, who works in MD Anderson Cancer Center, Houston, TX, USA, stressed that the IHC test, as well as identifying the lineage of the malignancy, helps us to quantify a crucial part in the management of oncological patients in this era of customised medicine from the expression levels of prognostic and predictive markers.

Quality control is a set of techniques used to detect, reduce and correct deficiencies in an analytical process, optimising each aspect of the IHC, from the arrival of the specimen to the issuing of the pathological report [44, 45].

The factors influencing the quality of the IHC include pre-analytical, analytical, and post-analytical factors. Among the pre-analytical factors, we have the tissue fixation, cold ischaemia time, the type of fixative used, the type of tissue and the type of processing, the use of other substances, such as descalers. The analytical factors include the type of primary antibody, the type of antigen retrieval, antibody dilution, detection system, the use of an automated or manual system, the type of positive and negative controls, and the training of medical technology staff, whereas the post-analytical factors consist of pathologist training to evaluate and report the presence or absence of the antigen [46].

The advantages and disadvantages must be evaluated when choosing between monoclonal, polyclonal antibodies and 'ready-to-use' antibodies. We can start by selecting the type of antigen retrieval; then, several primary antibody dilutions must be evaluated to see which is the one that generates the best ‘signal to noise ratio’. Another critical point is to choose the positive and negative control [47, 48] and document the conclusions in a laboratory protocol.

Finally, clinical evaluation ensures that the trial works as expected for clinical use. Therefore, using at least 20 samples, 10 positive and 10 negative, is recommended to evaluate the concordance between the obtained and expected results. IHC quality control must be conducted regularly and have a protocol to follow when there are failures in the process and an action plan [49].

### • Interventional cytology as a strategy in public health

Dr. Ricardo H Bardales, who works in Outpatient Pathology Associates/Precision Pathology, Estados Unidos, highlighted that fine-needle aspiration biopsy (FNAB) is an effective weapon in the diagnosis of palpable lesions, including cancer, and he stressed that ideally, it is the doctor (preferably pathologist) who interviews and examines the patient does the FNAB and interprets the sample to correlate cytological findings with clinical impression. To perform the procedure, a 25-gauge needle must be used without using suction (by capillarity). In that way, the unsatisfactory samples are reduced by utilizing a good clinical examination, appropriate technique, and extended and quality staining [50].

Dr. Bardales endorses the above outcomes in the breast cancer screening programme using FNAB. This programme was directed by the Norwegian Cancer Society and developed rigorously in Trujillo-Peru between 2012 and 2016. They found 347 breast abnormalities on physical examination; therefore, 159 FNAB were performed, where 11 cases provided a positive diagnosis for carcinoma or atypia, and the most common benign lesion was mastitis [51]. He stated that more recently, he has been using FNAB without aspiration in a retrospective study of 332 patients, in another prospective study during the COVID-19 pandemic, and in a third study as an intraoperative evaluation, replacing the frozen biopsy from 1,712 patients has obtained sensitivity, specificity and accurate diagnosis >90% [52]. On the economic side, Dr. Bardales said that FNAB in the US costs under $1,000 compared to excisional biopsy or core biopsy, which varies between $2,000 and $8,000 on top of hospital charges.

Finally, he mentioned that the advantages of FNAB include: Being a walk-in process, well tolerated, without significant complications, at a low cost, and high diagnostic accuracy, which enables fewer unnecessary surgeries. For the speaker, FNAB in skilled hands provides a highly accurate and rapid diagnosis, helpful to proceed with appropriate treatment, be it clinical or surgical, and that could replace a large number of surgical biopsies. FNAB is also a triage weapon to obtain samples for cultures, flow cytometry, hormonal dosages (parathormone, thyroglobulin, calcitonin), and molecular studies.

### Cytology and diagnosis of lung carcinoma on small biopsy

Dr. Ricardo Lastra, who works in The University of Chicago Medical Center, Estados Unidos, contextualised lung carcinoma as one of the world's most common malignancies, for which traditional treatment options have not managed to prolong overall survival. Fortunately, he noted that the progress in targeted therapy and immunotherapy has improved the outcome of metastatic non-small cell carcinomas, which are now considered the standard treatment when actionable mutations are identified.

Lung carcinoma diagnosis and staging require cytology samples or small transbronchial biopsies. For Dr. Lastra, pathology is therefore responsible for maximising the use of samples to diagnose and conduct molecular studies and predictive biomarker staining for patient management.

Recommendations have been issued on efficiently using small lung samples [53–56]. If the cytohistological characteristics of the tumour are diagnosed by histotype, immunohistochemical staining is not required. However, a limited IHC panel, including TTF-1 and p40, is often necessary to differentiate adenocarcinomas from squamous cell carcinomas. Neuroendocrine markers should be used if there is evidence of neuroendocrine differentiation. At present, reserve cut-offs are required for PD-L1 IHC and/or EML4-ALK FISH. The use of the sample can be maximised by dividing the tissue received for macroscopic study into more than one block.

FFPE samples, as well as air-dried cytology swabs, are reliable sources of DNA. Rapid on-site evaluation can immediately advise if the material is suitable or unsuitable for molecular studies. A fixative and mounting media that will not negatively affect the DNA should be selected [57].

In summary, Dr. Lastra noted that the pathology department is responsible for determining the methodology and workflow to maximise the use of these small samples. As such, the patient and clinical team can be given relevant information, thus avoiding additional biopsies due to inadequate samples.

### Preanalytical phase in breast cancer

During his presentation, Dr. Henry Guerra, who works in INEN, Peru, emphasised the importance of the preanalytical phase in pathology procedures, especially in breast cancer.

He pointed out that there is a focus on an innovation-based and constant improvement approach to specific methodologies pertaining to the histological analysis process and additional testing, such as IHC and molecular testing. However, the preanalytical phase is underestimated because it is considered the most uncomplicated phase in the histological process. The relevant protocols and recommendations should be followed, including ischaemic time and appropriate mounting media for the assessed sample. These are necessary for reliable results to be obtained. This is gaining importance now that more modern, updated, and sophisticated analytical methodologies are becoming available. We will only get results with dubious credibility and poor quality if the preanalytical phase is not implemented correctly.

## Conclusions

The seventh session of the JoPaO, which was held virtually, helped bring us closer to the health professionals who could not attend in person owing to the pandemic. Also, using a virtual platform facilitated discussions with world-class speakers from all over the world. They each provided excellent presentations and shared their experience from a Latin American perspective.

As highlighted by these speakers, the recent approval of the Peruvian cancer law creates new opportunities to transform the country's oncology services. Of particular relevance is the promotion of the National Network of Oncology, which seeks to coordinate and organise efforts to improve the journey for patients and decentralise health services. Another critical element of this legislation is the health technology evaluation, facilitating access to innovative therapies under efficient recruitment mechanisms. A particular focus was put on cancer epidemiological monitoring and creating a tumour bank, which seeks to put Peru at the forefront of the fight against cancer.

Cancer is a social and economic issue and not simply a medical matter. Despite creating a new legal framework in Peru, because our health system is fragmented and centralised it remains fragile. It is, thereby, imperative to develop strategic plans in which the foundations of research and continuous improvement take the social, cultural, economic, and geographic differences in our country into consideration.

The search for quality in oncological pathology makes it possible to reduce the mistakes that could arise in any of these phases. Those that occur in the preanalytical phase are particularly important. The impact and benefits of adequate quality control have been demonstrated in the CaCU Control Programme in Chile, ultimately improving cancer patients' survival.

A medical pathologist plays a central role in care plans for patients with cancer. They are vital in managing the often smaller samples, such as fine needle aspirates or small tissue fragments. This may reduce not only the time but also the cost. Their active participation in multidisciplinary teams and oncology public health policies is indisputable.

## List of abbreviations

FNAB, Fine-needle aspiration biopsy; CC, Cervical cancer; FFPE, Formalin-fixed paraffin-embedded; H&E, Haematoxylin and eosin; HSIL, High-grade squamous intraepithelial lesion; LMIC, Low- and middle-income countries; IHC, Immunohistochemistry; INEN, National Institute of Neoplastic Diseases; JoPaO, Oncological Pathology Conference; MINCYT, Ministry of Science, Research, and Technological Innovation; PAP, Pap smear test; HPV, Human papillomavirus; HR-HPV, High-risk human papillomavirus.

## Conflicts of interest

The authors declare that there are no conflicts of interest.

## Funding

The authors declare no funding was received from any public or private organisation.
